# Healthcare utilization patterns prior to a first heart failure diagnosis in a 90 day mortality cohort: A retrospective cohort study

**DOI:** 10.1016/j.ijcrp.2026.200678

**Published:** 2026-07-04

**Authors:** Ellinore Nilsson, Louise Pettersson, Björn Agvall

**Affiliations:** a Halland Hospital Halmstad, Region Halland, Halmstad, Sweden; b Department of Research and Development, Region Halland, Halmstad, Sweden; c Department of Clinical Sciences, Division of Pathology, Lund University, Lund, Sweden; d Center for Primary Health Care Research, Department of Clinical Sciences, Lund University, Malmö, Sweden

**Keywords:** Heart failure, Mortality, Healthcare-seeking behavior, Healthcare utilization, Primary care

## Abstract

**Background:**

Early recognition of emerging heart failure prior to diagnosis could offer an opportunity for prevention-focused care, but it remains unclear whether healthcare seeking patterns before diagnosis indicate an earlier window for intervention. The objective was to determine if changes in utilization appeared during the 12-week pre-diagnosis period preceding a first diagnosis of heart failure with poor short-term prognosis.

**Methods:**

A retrospective population-based study in Region Halland, Sweden, used the Regional Healthcare Information Platform to identify persons aged 40-90 years with incident heart failure during 2013 to 2019 who had died within 90 days. Encounters across inpatient and outpatient care settings, estimated glomerular filtration rate, N-terminal pro B type natriuretic peptide (NT-proBNP), echocardiography status and comorbidities were retrieved. Repeated measures analysis assessed trends in weekly visits during the 12-week lookback, with covariance analysis adjusted for age, sex, hypertension, ischemic heart disease and diabetes.

**Results:**

The cohort included 902 individuals, mean age 85 years, 52% women. Echocardiography was not registered in 65%. Mean NT-proBNP measured 11,210 ng/L. The total average number of visits per week increased from 0.42 at week minus 12 to 1.77 at week 0 (p < 0.001). Primary care visits had increased from 0.30 to 0.72 (p < 0.001). Adjustments did not alter the pattern. No consistent early rise in visits appeared before the final two weeks.

**Conclusions:**

In this high-mortality population, healthcare utilization remained low and unchanged until a sharp rise immediately before diagnosis, indicating a narrow and late window for preventive action, which limits the feasibility of preventive intervention in routine care.

## Highlights

• Examined healthcare utilization patterns in patients dying within 90 days of a first HF diagnosis.
• The cohort had high NT-proBNP (11,210 ng/L), high CCI >5, and 65% without echocardiography.
• Visit frequency remained low and stable until a sharp rise 1–2 weeks before diagnosis.
• No early warning pattern of healthcare encounters seen in the 12-week pre-diagnosis period.

## 1 Introduction

Heart failure affects approximately 2% of the population and is associated with high morbidity and mortality [[1], [2], [3]]. Its prevalence escalates with age, reaching 11% in men and 14% in women ≥80 years old [4]. Heart failure entails substantial impairment in quality of life due to extensive physical and psychological symptom burden and is associated with high morbidity, including frequent hospitalizations and significant healthcare utilization [5,6]. A previous study in Region Halland showed that patients with heart failure had an annual mortality of 16% [2]. Hospital-based studies often report higher mortality rates up to 21% annually due to the inclusion of patients with more unstable heart failure compared to outpatient populations, who are considered to have a more stable heart failure, where mortality ranges between 6 and 7% [1,2,[7], [8], [9]]. Approximately 44% of heart failure patients are admitted to internal medicine departments at least once a year whereas 18% are readmitted within 30 days [8,10]. Heart failure is associated with a high hospitalization burden, with frequent readmissions and substantial inpatient resource use. Contemporary real-world studies demonstrate high annual readmission rates and multiday cumulative hospital stays per patient year, consistent with an average of approximately 6–7 inpatient days annually [2,8,10,11]. Previous studies indicated that about one-third of patients are readmitted for cardiovascular reasons within 100 days, and one-fifth specifically for heart failure [[12], [13], [14]]. Clinical factors present at discharge, for example advanced age, renal impairment, elevated NT-proBNP, are strongly associated with the risk of readmission and are often detectable before the patient leaves the hospital.

The highest mortality rates in heart failure mainly occur in the period following discharge, particularly within the first 30–100 days, as highlighted in prior research [13,14]. Moreover, a significant proportion of patients who die shortly after a heart failure diagnosis have not undergone echocardiography, with these cases predominantly identified within hospital settings [13,15].

A Swedish study that analyzed patients diagnosed with prostate, breast, colorectal, lung, gynecological, and skin cancers, including malignant melanoma, in the western region of Sweden observed an increase in primary care consultations during the 50–100 days before their cancer diagnosis [16]. The study found that 56% of these patients visited a general practitioner more than four times in the year before their diagnosis [17]. Similarly, a Danish study observed a rise in primary care consultations 4-6 months prior to a cancer diagnosis, regardless of the patients' usual consultation frequency [18]. Previous studies from southwest Sweden have highlighted the high mortality risk associated with heart failure, with up to 15% of individuals dying within 90 days of diagnosis [19]. This group, characterized by advanced age and comorbidities, is identified as having a particularly high risk of mortality shortly after diagnosis [15,20]. Previous research has suggested that increasing healthcare utilization may precede clinical recognition of heart failure. In a population based study of incident acute heart failure hospitalization, healthcare contacts increased progressively during the year before admission, with a more pronounced rise beginning approximately 6 to 8 months prior [21]. However, it remains uncertain whether there are identifiable warning signs preceding the onset of heart failure, and whether individuals seek care for early symptoms before diagnosis or are already on the brink of requiring hospitalization, particularly in older, multimorbid populations with poor short term prognosis.

The objective of this study was to investigate occurrence of changes in the frequency of consultations prior to the onset of severe heart failure among individuals who died within 90 days of diagnosis. Specifically, the study aimed to explore whether an increased number of primary care visits before the onset of heart failure could serve as an early warning sign, potentially indicating individuals at high risk for rapid deterioration.

## 2 Method

This retrospective observational study analyzed a heart failure population in Region Halland, Sweden, using registry data from the Regional Healthcare Information Platform (RHIP). Region Halland has a population of 340,000 and a healthcare infrastructure comprising 3 hospitals, 40 inpatient wards, 2 emergency departments, 30 specialist outpatient clinics, and 46 primary care clinics. Of these primary care units, 23 were privately operated, while 23 were publicly administered.

### 2.1 Data source

Region Halland utilizes the RHIP database, which provides pseudo-anonymized data that includes clinical, operational, and financial information for all individuals treated since 2011 in both public and private healthcare facilities within the region. This study relied on RHIP as its primary data source, a method previously used in research on heart failure populations in Region Halland [2,13,15,19]. RHIP consolidated data from various healthcare services, including primary care, emergency services, hospital admissions, outpatient, and inpatient care [22]. It offered a comprehensive overview of the patient population in Region Halland, linking clinical, operational, and cost data at the level of individual encounters and including details on healthcare system resources and capacity, such as the availability of nurses, doctors, and hospital beds. RHIP records contained information on deceased patients, including the date of death, which facilitated the analysis of overall mortality within the cohort. Information on prescribed medications was obtained from the National Pharmacy Register.

### 2.2 Study procedure

This study included patients aged 40 to 90 years who were diagnosed with heart failure in Region Halland. The data analyzed spanned from 2013 to 2019, focusing on individuals who died within 90 days of their heart failure diagnosis. All individuals included had an incident heart failure diagnosis, defined as the first registered heart failure diagnosis during the study period. No patients had a documented prior heart failure diagnosis. Severity was defined by the clinical presentation at diagnosis and the subsequent outcome of death within 90 days, not by disease duration or chronic stage. The cohort was intentionally restricted to individuals who died from any cause within 90 days after incident HF diagnosis. Heart failure was not required to be the primary cause of death, and cause-specific mortality data were not available. This restriction was applied to capture severe initial presentations with extremely poor short-term prognosis and to assess whether healthcare-seeking behavior prior to diagnosis shows any warning signals in this clinically critical subgroup. The criteria for diagnosing heart failure and comorbidities are detailed in Supplementary Table 1. The study period commenced at the time of the first diagnosis (the index date) and there was a 90-day lookback period prior to the onset of heart failure for these patients.

Laboratory data from index, including estimated glomerular filtration rate (eGFR) and N-terminal pro b-type Natriuretic Peptide (NT-proBNP) were captured. To account for acute conditions that may influence NT-proBNP concentrations and short-term prognosis, secondary diagnoses recorded in temporal proximity to the incident heart failure diagnosis were identified. ICD-10 codes were used to capture acute coronary syndrome (I21, I24, I20.0), pulmonary embolism (I26), acute kidney disease (N17), myocarditis or endocarditis (I40, I33), and sepsis or systemic infection (A40, A41, R57.2, R65.0, R65.1). Diagnoses were included if registered from one week before to two weeks after the index date. The study monitored healthcare utilization by recording the number of hospital admissions and the total days of hospitalization for the lookback period. The number of visits to nurses and physicians in outpatient settings, such as hospital outpatient departments, primary care facilities, and emergency departments, was documented for in the lookback period. Pharmacological treatments at index were registered for betablockers, renin-angiotensin-system inhibitors (RASi), mineralocorticoid receptor antagonist (MRA) and loop diuretics with the relevant ATC codes provided in Supplementary Table 2.

The Charlson Comorbidity Index (CCI) was recorded and assessed at index and data on comorbidities including hypertension, ischemic heart disease, cerebrovascular stroke, atrial fibrillation, diabetes, chronic obstructive pulmonary disease, dementia, and malignancies were also obtained [23]. The CCI quantifies comorbidity burden by assigning weighted scores to 17 conditions based on their mortality risk. Each condition is scored 1, 2, 3, or 6, and the total score is obtained by summing these values. In this study, the CCI was calculated at the time of heart failure onset, designated as the index date.

### 2.3 Statistics

Descriptive statistics were used to summarize the data. Continuous variables are presented as means with standard deviations (SD), and differences between groups were assessed using the student's t-test. Categorical variables were analyzed using the Chi-square test, with frequencies and percentages reported.

NT-proBNP levels were measured at the time of initial heart failure diagnosis, within a three-month window before the index date. When multiple NT-proBNP measurements were available, the highest value was selected as it was considered most representative of the patient's cardiac function. These values were categorized into three groups based on their association with heart failure: “HF unlikely” for normal NT-proBNP levels, and “grey zone” or “HF likely” for elevated levels, as described in Supplementary Table 2 [24,25].

Kidney function was assessed by the estimated glomerular filtration rate (eGFR) (ml/min/1.73 m^2^), measured within three months before diagnosis. The value closest to the index date was used for classification. Kidney function was categorized as normal (eGFR ≥60 ml/min), reduced (eGFR 30–59 ml/min), and impaired (eGFR <30 ml/min) [26].

To analyze the differences in the number of visits per week across the 12-week period (from week 12 before index to week 0 being at index), a repeated measures ANOVA was performed. The within-subject factor was time (13 levels, from week 12 to week 0 before the diagnosis date). Covariance analysis was employed adjusted for sex, age, hypertension, diabetes, ischemic heart disease. A predefined subgroup analysis was conducted excluding individuals with acute conditions recorded in temporal proximity to the heart failure diagnosis, including acute coronary syndrome, pulmonary embolism, acute kidney disease, myocarditis, endocarditis, and sepsis. Temporal patterns in healthcare utilization were analyzed in the same manner as in the overall cohort.

The total healthcare utilization during the 12-week lookback period was compared between individuals with and without acute conditions. Differences in mean number of visits were assessed using the Student's *t*-test.

All statistical analyses were two-tailed, with a significance threshold of p < 0.05. Analyses were conducted using IBM SPSS Statistics, Version 31.0, IBM Corp., Armonk, NY, USA.

## 3 Result

A total of 902 individuals met the criteria for further investigation, all of whom died within 90 days of the incident heart failure diagnosis. Patient characteristics are summarized in Table 1. Among the participants, 584 (65%) had from available data, no registered echocardiography prior to their death. Among those who did, 120 (13%) were diagnosed with HFpEF, 73 (8%) with HFmrEF, and 125 (14%) with HFrEF. The median age of the cohort was 85 years, with a gender distribution of 52% women and 48% men.

**Table 1:** Summary of the basic characteristics of the study population, including demographic information, clinical features, and comorbidities.

	Total
Number of patients	902
Age, mean (SD)	85 (9)
*Gender*	
Women, n (%)	467 (52)
Men, n (%)	435 (48)
*Renal function*	
eGFR ml/min, mean (SD)	45 (21)
eGFR >60, n (%)	252 (28)
eGFR 30-60, n (%)	405 (45)
eGFR <30, n (%)	245 (27)
*Natriuretic peptides*	
NT-proBNP ng/l, mean (SD)	11210 (15406)
HF unlikely, n (%)	41 (6)
Grey zone, n (%)	129 (19)
HF likely, n (%)	506 (75)
*Comorbidities*	
Hypertension, n (%)	652 (72)
IHD, n (%)	373 (41)
CVI, n (%)	214 (24)
Atrial fibrillation, n (%)	401 (44)
Diabetes, n (%)	228 (25)
COPD, n (%)	156 (17)
Dementia, n (%)	255 (28)
Malignancy, n (%)	168 (19)
*Pharmacotherapies*	
Betablockers, n (%)	475 (53)
RASi, n (%)	353 (39)
MRA, n (%)	88 (10)
Diuretics, n (%)	404 (45)
Digoxin, n (%)	50 (6)

Note = HF= Heart Failure, SD=Standard Deviation, eGFR= Estimated Glomerular Filtration Rate, NT-proBNP= N-terminal pro B-type Natriuretic Peptide, HF unlikely = Heart Failure unlikely, HF likely = Heart Failure likely, IHD= Ischemic Heart Disease, CVI= Cerebrovascular Insult, COPD= Chronic Obstructive Pulmonary Disease, RASi= Renin-Angiotensin System inhibitors, MRA = Mineralocorticoid Receptor Antagonists.

Acute conditions potentially contributing to elevated NT-proBNP concentrations were identified in a subset of individuals. Acute coronary syndrome was recorded in 172 individuals (19%), acute kidney disease in 138 (15%), sepsis or systemic infection in 66 (7%), pulmonary embolism in 34 (4%), and myocarditis or endocarditis in 10 (1%) within the predefined time window. Acute kidney disease was based on ICD-10 coding and interpreted alongside renal function measurements, with 27% showing estimated glomerular filtration rate below 30 ml/min at diagnosis. Notably, 567 individuals (63%) had none of these accompanying acute conditions recorded.

Pharmacological treatments reflect medication use prior to the incident heart failure diagnosis. The therapies were prescribed before heart failure recognition, when phenotype and left ventricular ejection fraction were not yet established.

**Fig. 1:**
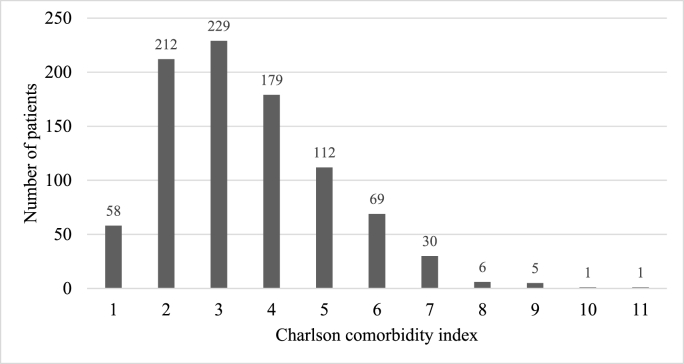
Distribution of the Charlson Comorbidity Score across the entire study population.

CCI results show that 678 (75%) individuals had a low CCI, defined as <5 points. The distribution of the Charlson Comorbidity Score is illustrated in Fig. 1.

During the 12-week lookback period, patients had an average of 5.5 inpatient days each. The average number of visits to the emergency department was 1.2 visits during this period.

**Fig. 2:**
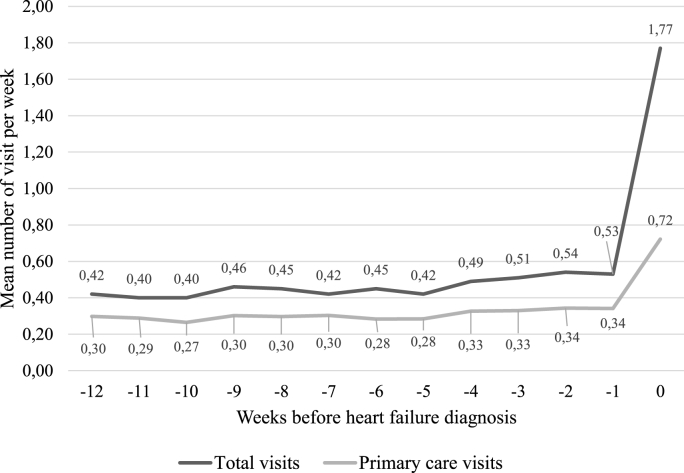
Trend in the average number of visits per week during the 12-week lookback period leading up to the diagnosis of heart failure.

Repeated measures ANOVA revealed a significant difference throughout the study period. Adjustments for sex, age, hypertension, ischemic heart disease, and diabetes were made by ANCOVA, but none of these factors did significantly affect the average number of visits. Fig. 2 shows the average number of visits per patient, both overall and in primary care, for each week leading up to the diagnosis of heart failure. The total average number of visits per week increased from 0.42 in week 12 to 1.77 (p < 0.001) in the week before diagnosis. In primary care, the average number of visits rose from 0.30 to 0.72 (p < 0.001) during the same period.

There is no significant variation in the average number of primary care visits from week to week until one week prior to the diagnosis, when the number increases from 0.34 to 0.72 visits per individual (p < 0.001). A similar trend is observed in the total average number of visits, which rises significantly from 0.53 to 1.77 visits per individual (p < 0.001). A notable increase in total visits is observed between weeks 5 and 4 before the diagnosis of heart failure. No clinically relevant differences were observed between women and men with regard to healthcare utilization patterns or mortality. A subgroup analysis excluding individuals with acute conditions showed similar temporal patterns in healthcare utilization, with no early increase in visits prior to diagnosis (Supplementary Fig. 1). No significant differences in cumulative healthcare utilization were observed between individuals with and without acute conditions. The mean total number of visits was 7.8 vs 6.9 (p = 0.60), and primary care visits 4.6 vs 4.3 (p = 0.30), respectively.

## 4 Discussion

This study investigated healthcare utilization patterns leading up to a heart failure diagnosis in individuals who died within 90 days of diagnosis, categorizing them as high-risk for mortality. The cohort was primarily elderly with a CCI ≤5, and two thirds had no registered echocardiography at diagnosis. Over the 12-week lookback period, the average number of healthcare visits increased from 0.42 visits per week at week −12 to 1.77 in the week before diagnosis, and primary care visits increased from 0.30 to 0.72. No gradual rise in visit frequency was observed; the increase appeared only in the final week before diagnosis, suggesting that visit frequency alone is not an early warning indicator.

The term severe heart failure in this study refers to the initial clinical presentation associated with extremely poor short-term prognosis, rather than to chronic advanced heart failure. Although these patients exhibited features consistent with advanced disease at diagnosis, all cases represented incident heart failure. The findings therefore reflect late detection or abrupt clinical deterioration at first presentation, rather than expected utilization patterns in patients with established advanced heart failure. The patient cohort primarily consisted of elderly individuals with a low CCI, although the overall clinical burden remained substantial due to advanced age and multimorbidity. The average age of participants was 85 years, which was higher than other studies having an average of approximately 77-78 years [1,3,4,23,27]. Since this cohort was considered as a high-risk mortality cohort, this could be expected. In patients with high comorbidity burden and limited diagnostic workup, natriuretic peptides may represent one of the few readily available indicators of advanced cardiovascular stress and imminent deterioration. The exceptionally high NT-proBNP level of 11,210 ng/L observed at diagnosis in this high-mortality population is greater than the mean levels reported in unselected populations, clearly indicating that the patients were severely ill [19,24]. At such markedly elevated concentrations, NT-proBNP reflects pronounced cardiac wall stress, consistent with probable advanced heart failure decompensation and substantial myocardial strain. However, despite their severe condition, only 35% had undergone echocardiography to objectively confirm the heart failure diagnosis prior to their deaths. The low echocardiography rate likely reflects advanced age, high comorbidity burden, and rapid clinical deterioration in this cohort, which may limit the feasibility and clinical relevance of comprehensive diagnostic evaluation. In addition, access and prioritization in real world practice, particularly outside specialized care, may contribute to the observed findings.

A further important interpretation of the present findings is that they may indicate delayed cardiological evaluation in a subgroup of elderly patients with high comorbidity burden, kidney impairment, and markedly elevated NT proBNP levels. This contrasts with prior studies reporting gradual increases in healthcare contacts before acute heart failure hospitalization, which did not include markers such as NT proBNP levels or renal function [22]. In this high mortality cohort, no early warning signal was observed in the frequency of healthcare utilization, whereas indicators of advanced disease were evident at presentation, including elevated NT proBNP and prevalent renal impairment. Although many individuals in this cohort had limited overall prognosis driven by non-cardiac conditions, the findings raise concern that potentially relevant cardiac pathology may not always have been assessed in a timely manner, particularly given that only 35% underwent echocardiographic evaluation. In selected patients, earlier reassessment, including echocardiography and referral to cardiology specialist care, may have been associated with clinically meaningful differences. Markedly elevated NT proBNP may therefore be regarded as a signal of significant cardiovascular stress that warrants consideration of careful clinical evaluation, while clinical decision making must consider frailty, comorbidity burden, and overall goals of care. Although acute conditions such as sepsis, acute kidney disease, pulmonary embolism, and acute coronary syndromes can contribute to elevated NT-proBNP concentrations, nearly two thirds of individuals had no documented accompanying acute diagnosis in temporal proximity to heart failure onset. This supports that the markedly elevated natriuretic peptide levels in this cohort primarily reflected severe cardiac decompensation at first presentation rather than alternative acute conditions. The prevalence of non-cardiac comorbidities was lower in present study than in hospital-based heart failure registries, where approximately 80% of patients have at least one comorbidity. This likely reflects registry selection of cardiology-assessed hospitalized patients, whereas the present study is population-based and includes incident heart failure across all care settings [27].

CCI is commonly used to predict mortality risk, and in cancer populations, a CCI score >3 is often associated with a poor prognosis. For example, previous studies of prostate cancer patients showed that CCI >1 correlated with higher all-cause and other-cause mortality, although it did not impact cancer-specific mortality [18]. In our cohort, the high comorbidity burden likely reflects the advanced age and multiple health conditions prevalent among these individuals. While CCI has not been shown to predict long-term mortality in elderly individuals with heart failure in previous studies, a comorbidity burden (CCI >2) at the time of a first hospitalization for acute heart failure has been identified as an independent predictor of mid-term post-discharge mortality in this population [28,29].

One of the key findings in this study was the increasing number of healthcare visits as the patient's approached onset of heart failure diagnosis. Over the 12-week lookback period, the total average number of visits per week rose significantly, from 0.42 visits in week −12 to 1.77 (p < 0.001) visits in the week prior to diagnosis. Primary care visits also increased over this period, though to a lesser extent, from 0.30 to 0.72 (p < 0.001) visits per week. These findings suggest that the lack of early warning signals in healthcare utilization is inherent to this patient group rather than driven by identifiable acute conditions as triggers. In contrast to several other chronic diseases, where increased healthcare contact may precede diagnosis, this highlights the limited value of care-seeking behavior as an early detection tool for incident heart failure. Cancer studies show earlier increases in primary care consultations, occurring about 50 days before breast and gynecological cancers and 80 to 100 days before prostate and lung cancers [17,18]. Such patterns were not seen in present study. Instead, the findings indicate that even in this severely ill population, early detectable changes in healthcare-seeking behavior were limited. No sex-based differences were observed in healthcare utilization patterns or short-term outcomes, indicating that the adverse prognosis in this cohort was comparable in women and men. This underscores that advanced age, comorbidity burden, and clinical presentation outweighed sex related factors in determining outcome.

Previous work reported a gradual rise in healthcare contacts several months before first heart failure hospitalization, suggesting potential opportunities for earlier diagnosis [21]. In contrast, no early increase was observed in the present cohort, with healthcare utilization remaining stable until a sharp rise immediately before diagnosis. This difference likely reflects variation in study populations, as the present cohort was older and restricted to individuals who died within 90 days, representing a high mortality group with advanced disease. Age may further contribute, as younger individuals have been reported to show steeper increases in healthcare contacts prior to hospitalization. In addition, the present cohort was characterized by markers of advanced disease, including a high prevalence of renal impairment, markedly elevated NT proBNP levels, and limited use of echocardiography, variables not included in earlier analyses. The more granular weekly assessment indicates that increases in healthcare contacts occurred only in close proximity to diagnosis.

The sharp rise in visits during the last two weeks before a heart failure diagnosis suggests that worsening symptoms may occur abruptly, prompting patients to seek care at primary care or emergency department closer to the time of their initial diagnosis and no early warning signs were identified in the data leading up to the diagnosis. Delays in seeking care for heart failure symptoms can range from a few hours to several days between symptom onset and hospital admission [30]. Research indicates that more than 10% of patients readmitted for heart failure postpone seeking treatment for over two days after symptoms begin [31]. Consequently, it seems that heart failure patients tend to seek care at least within a week before heart failure diagnosis. Despite the increase in care-seeking behavior in the final weeks before heart failure diagnosis, no clear, consistent warning signs were identified that could predict the imminent onset of heart failure in these severely ill patients which is consistent with previous studies [32,33]. This lack of early indicators makes it challenging to develop strategies for earlier detection.

Markedly elevated NT-proBNP levels at first presentation may represent a critical clinical signal of advanced cardiovascular stress and imminent risk. Such findings should alert physicians and nursing staff to a high-risk clinical situation and prompt immediate reassessment of management priorities. While expedited involvement of specialized heart failure services may be appropriate in selected cases, the very high short-term mortality observed in this cohort underscores the importance of early initiation or consideration of structured palliative care management. For many individuals with severe initial presentation, timely discussions regarding goals of care, symptom-oriented treatment, and end of life planning may be as clinically relevant as escalation of disease directed therapy. The absence of early changes in healthcare utilization suggests that alternative strategies beyond care-seeking patterns are needed for earlier identification.

### 4.1 Strengths and limitations

One of the strengths of this study is the large, population-based sample drawn from Region Halland, which provided comprehensive healthcare utilization data over an extended period. The use of the RHIP allowed for a detailed analysis of patient interactions with the healthcare system, including primary care, hospital admissions, and outpatient encounters. This longitudinal data is valuable for understanding care-seeking patterns in high-risk individuals. Additionally, the study's focus on patients who died within 90 days of heart failure diagnosis provides important insights into the care trajectory of patients with severe heart failure. This high-mortality subgroup was intentionally selected because they represent the patients in whom warning signs, if present, would be most crucial for prevention.

The fact that 65% of the cohort had not undergone echocardiography limits the ability to confirm heart failure diagnosis and assess its severity accurately. However, the low echocardiography rate is typical in patients presenting with terminal or near-terminal HF and reflects real-world diagnostic practice in this population. Heart failure is associated with a substantially adverse prognosis irrespective of left ventricular ejection fraction phenotype, particularly in elderly individuals with advanced age and high comorbidity burden. The high short-term mortality observed in this cohort therefore reflects severe clinical presentation rather than dependence on documented heart failure phenotype. The absence of echocardiographic assessment in a large proportion of patients likely reflects real world practice in individuals with rapid clinical deterioration, as reflected by the high short-term mortality observed in this cohort, and limited survival, rather than reduced disease severity. Furthermore, as the cause of death was not systematically recorded, it is possible that not all deaths in the cohort were directly attributable to heart failure. The study was also limited by its observational nature, which restricts the ability to draw causal conclusions. Moreover, the cohort's age (median 85 years) and high comorbidity burden mean that the findings may not be generalizable to younger or less complex populations. The study only included individuals from Region Halland, so the findings may not be applicable to other regions or countries with different healthcare systems.

SGLT-2 inhibitors were not included, as these agents were not established as heart failure therapy during the study period (2013–2019), and any use would have reflected treatment for diabetes rather than cardiovascular indications.

NT proBNP measurements were obtained from both outpatient and inpatient care. The database did not permit determination of which professional category-initiated test ordering, and attribution to cardiologists, specialized nurses, or general practitioners could therefore not be reliably assessed.

## 5 Conclusion

This study shows that in a high mortality heart failure population, healthcare utilization remained low until a sharp increase immediately before diagnosis, indicating a narrow and late window for preventive action. In contrast, markers of advanced disease, including elevated NT proBNP and renal impairment, were observed at presentation, suggesting that healthcare utilization alone may have limited value for early identification. The low rate of echocardiographic assessment indicates that these findings were not consistently accompanied by diagnostic evaluation. These results highlight the importance of considering clinical indicators of disease severity in addition to care seeking patterns when identifying patients at risk.

## Consent for publication

Not applicable.

## Ethics approval and consent to participate

This study was approved by the Swedish Ethical Review Board, Stockholm Department 2 Medicine, registration number 2020-00455. Informed consent was waived as per the board's approval. All methods followed applicable guidelines and regulations in accordance with the Declaration of Helsinki, with the waiver of consent process specifically sanctioned by the Swedish Ethical Review Board.

## Clinical trial number

Not applicable.

## Availability of data and materials

The datasets generated and analyzed in this study are not publicly available, in accordance with the Swedish Health and Medical Services Act and the Secrecy Act. However, these datasets may be accessible through Region Halland upon reasonable request to the corresponding author. Access will require review and approval by the Regional Consultative Committee for Data Collection in Region Halland.

## Funding

Not applicable.

## CRediT authorship contribution statement

**Ellinore Nilsson:** Conceptualization, Data curation, Formal analysis, Investigation, Methodology, Project administration, Validation, Visualization, Writing – original draft. **Louise Pettersson:** Conceptualization, Data curation, Formal analysis, Investigation, Methodology, Supervision, Validation, Visualization, Writing – original draft. **Björn Agvall:** Conceptualization, Data curation, Formal analysis, Investigation, Methodology, Project administration, Supervision, Validation, Visualization, Writing – original draft.

## Declaration of competing interest

The authors declare no conflict of interest.
