# Supplementary material for: Healthcare utilization patterns prior to a first heart failure diagnosis in a 90 day mortality cohort: A retrospective cohort study
Source: Int J Cardiol Cardiovasc Risk Prev. 2026 Jul 4;30:200678. doi: 10.1016/j.ijcrp.2026.200678 (PMC13380770; doi:10.1016/j.ijcrp.2026.200678)
Supplement: Multimedia component 1 [file mmc1.docx]

**Supplementary Information**

**Supplementary Table 1**

Supplementary Table 1. ICD-10 codes for heart failure and the comorbidities included in present study.

| **Variable** | **ICD-10 code(s)** |
| --- | --- |
| Heart failure | I50 |
| Hypertension | I10–I15 |
| Ischemic heart disease | I20–I25 |
| Cerebrovascular disease | I60–I69 |
| Atrial fibrillation/flutter | I48 |
| Diabetes mellitus | E10–E14 |
| Chronic obstructive pulmonary disease | J44 |
| Dementia | F00–F03, G30 |
| Malignancy | C00–C97 |
| Acute coronary syndrome* | I21, I24, I20.0 |
| Pulmonary embolism* | I26 |
| Acute kidney disease* | N17 |
| Myocarditis* | I40 |
| Endocarditis* | I33 |
| Sepsis/systemic infection* | A40, A41, R57.2, R65.0, R65.1 |

*Acute conditions assessed within one week before to two weeks after the incident heart failure diagnosis.

**Supplementary Table 2**

Supplementary-Table 2: List of evidence-based medications recommended for treatment of heart failure.

| **Medication (ATC-code)** |
| --- |
| Angiotensin-converting-enzyme inhibitors (C09) |
| Captopril |
| Enalapril |
| Lisinopril |
| Ramipril |
| Trandolapril |
| Betablockers (C07) |
| Bisoprolol |
| Carvedilol |
| Metoprolol |
| Nebivolol |
| Angiotensin receptor blockers (C09) |
| Candesartan |
| Valsartan |
| Losartan |
| Mineralocorticoid receptor antagonists (C03DA) |
| Eplerenone |
| Spironolactone |
