# Supplementary material for: Healthcare utilization patterns prior to a first heart failure diagnosis in a 90 day mortality cohort: A retrospective cohort study
Source: Int J Cardiol Cardiovasc Risk Prev. 2026 Jul 4;30:200678. doi: 10.1016/j.ijcrp.2026.200678 (PMC13380770; doi:10.1016/j.ijcrp.2026.200678)
Supplement: Multimedia component 2 [file mmc2.pdf]

Supplementary Figure 1. Temporal patterns in healthcare utilization prior to incident heart failure diagnosis stratified by presence of acute conditions.

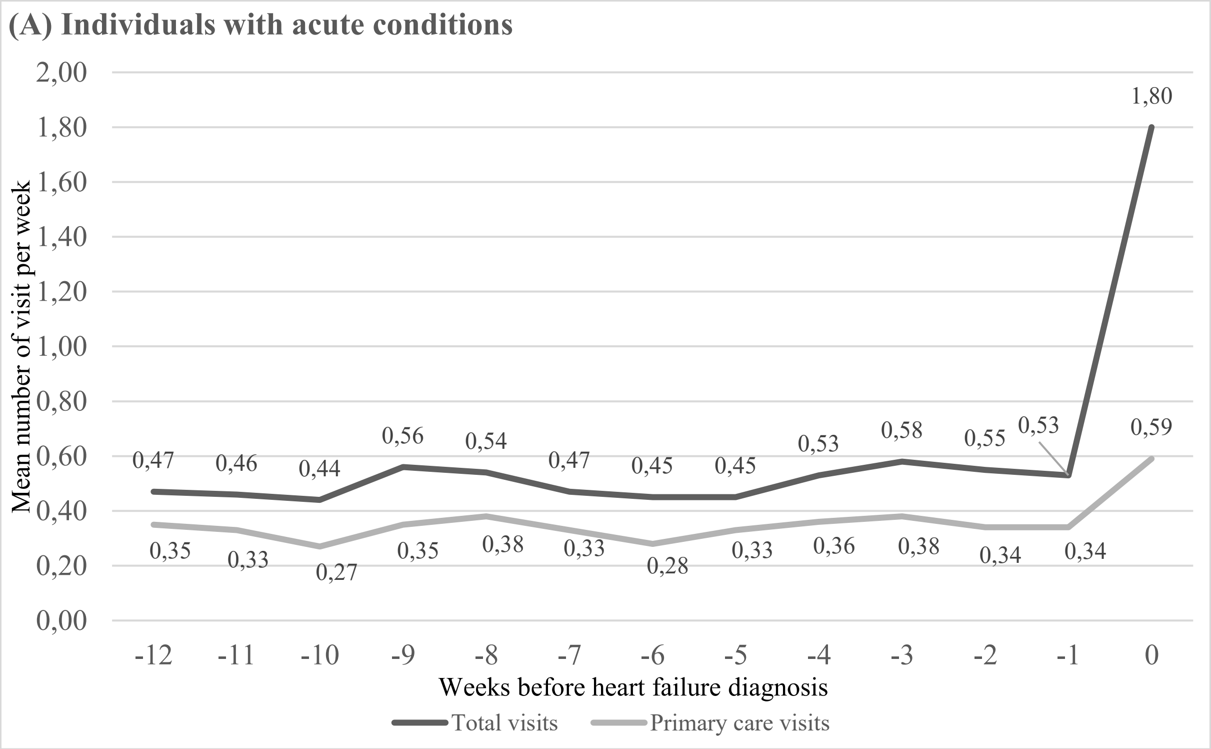

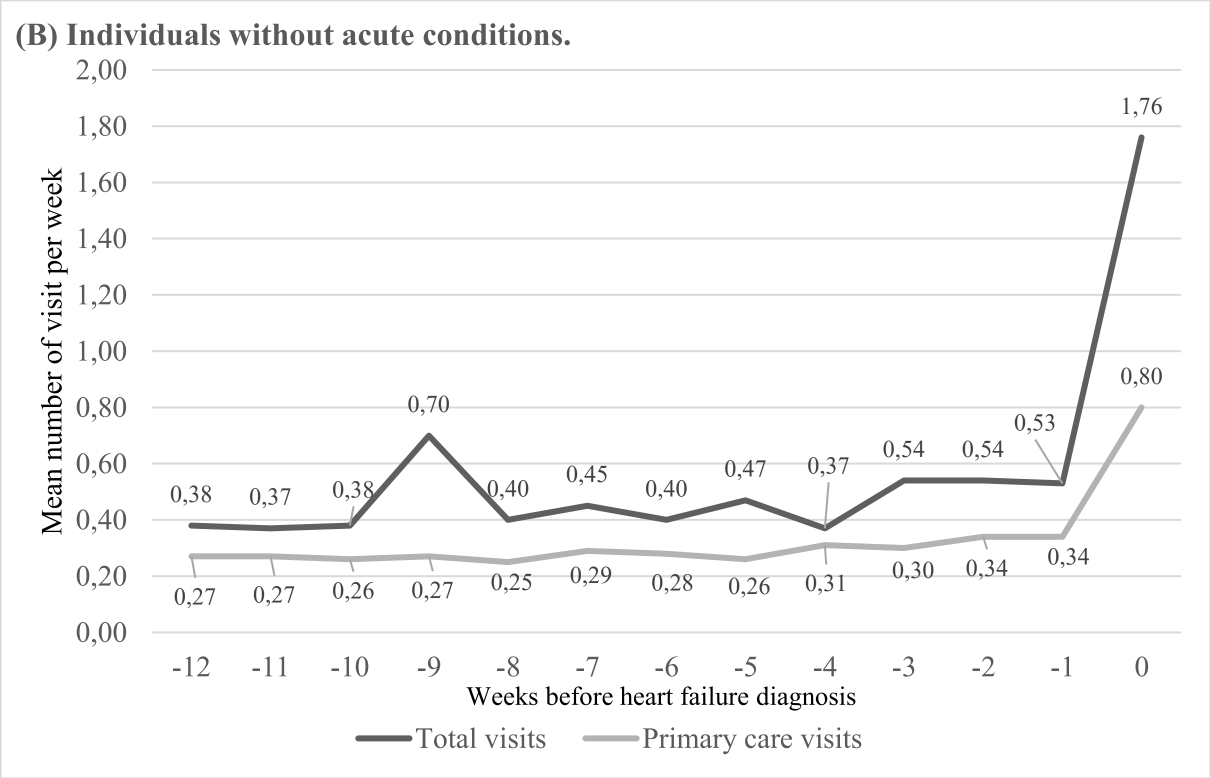

Note: (A) With acute conditions. (B) Without acute conditions.
Mean weekly number of total and primary care visits during the 12-week lookback period (week −12 to week 0).
